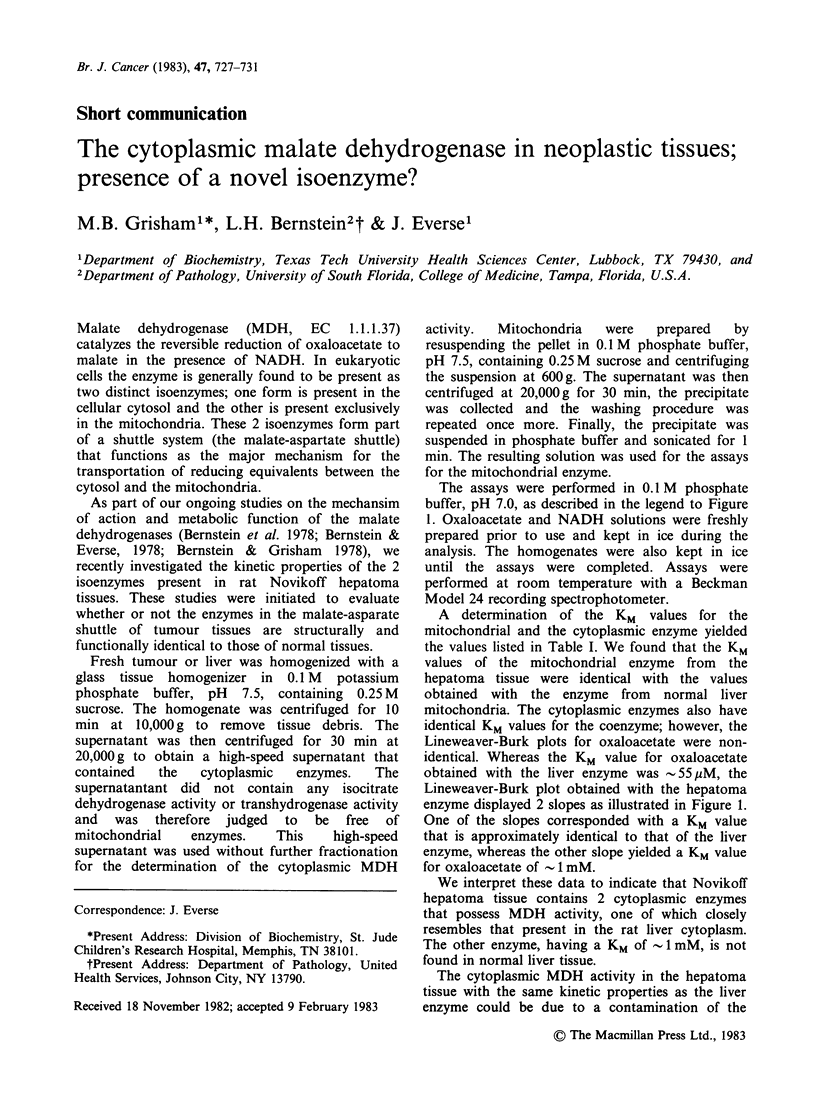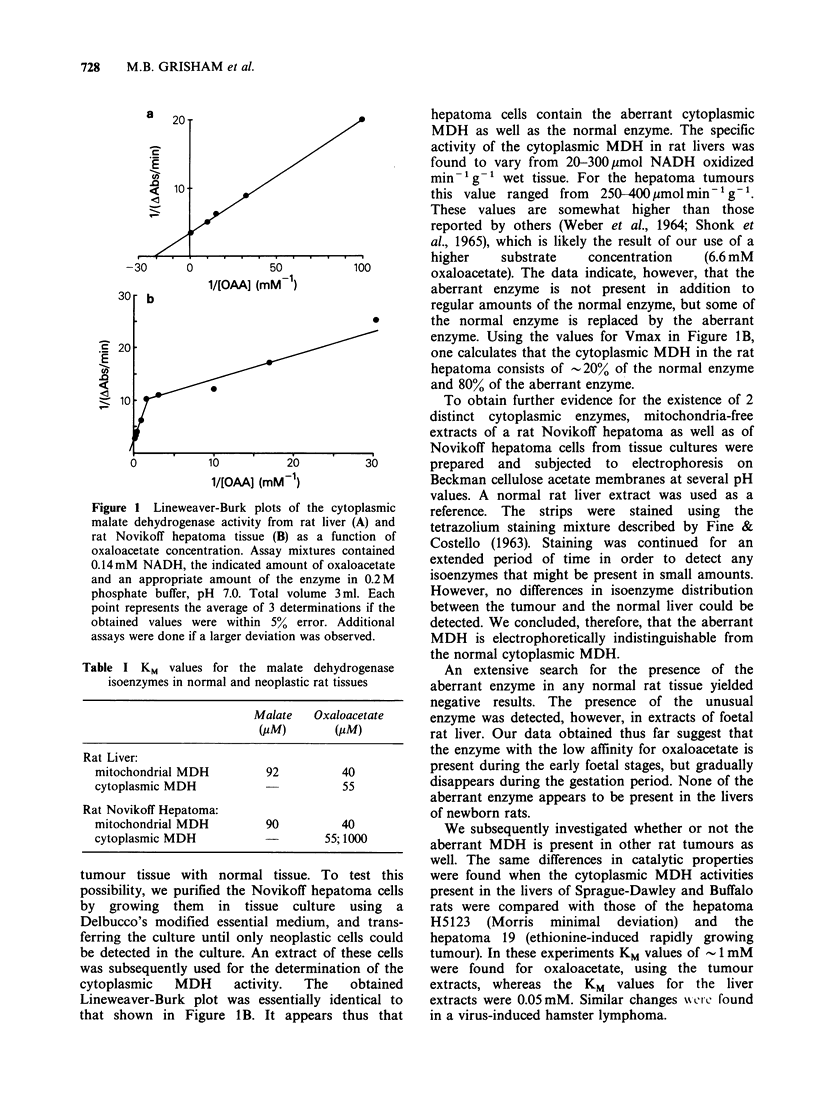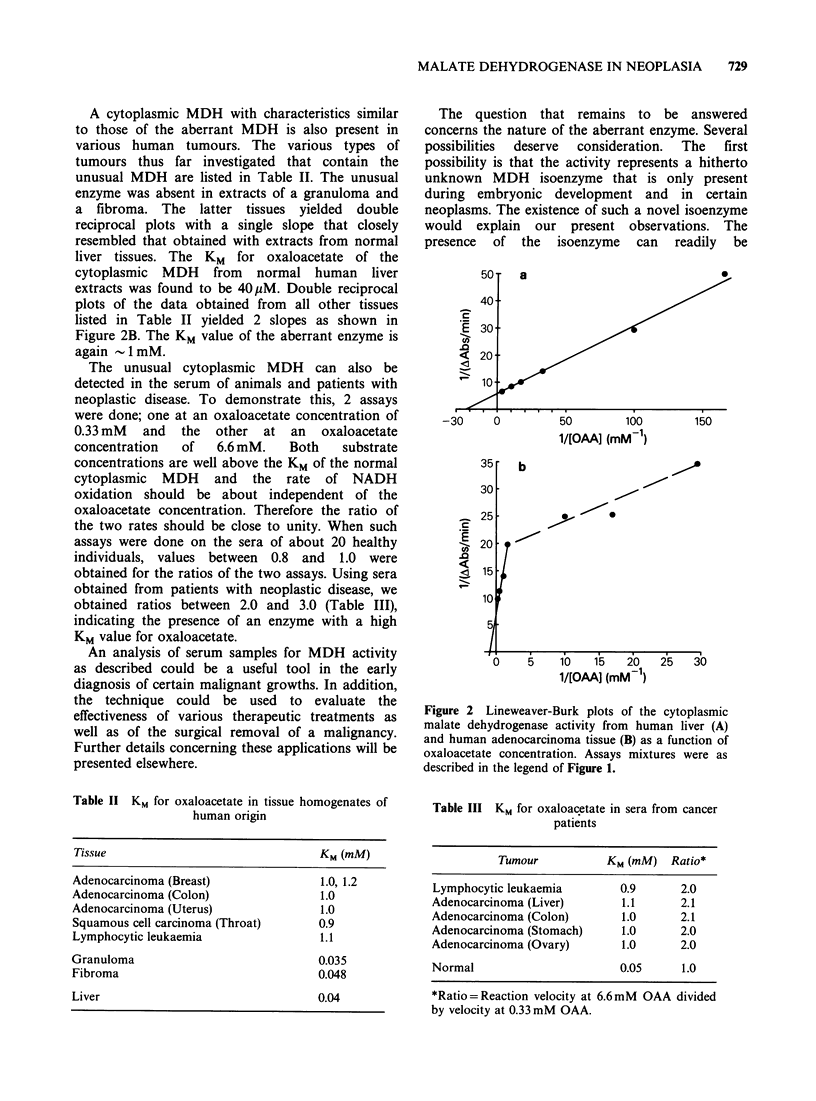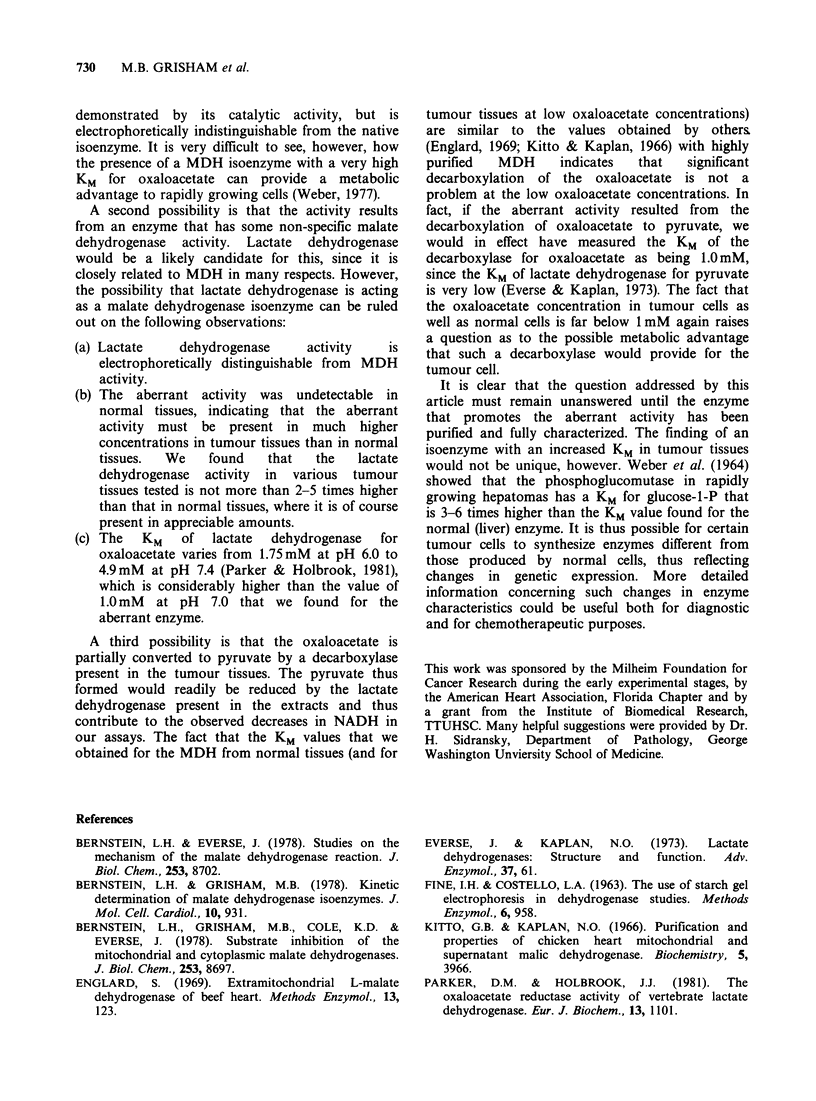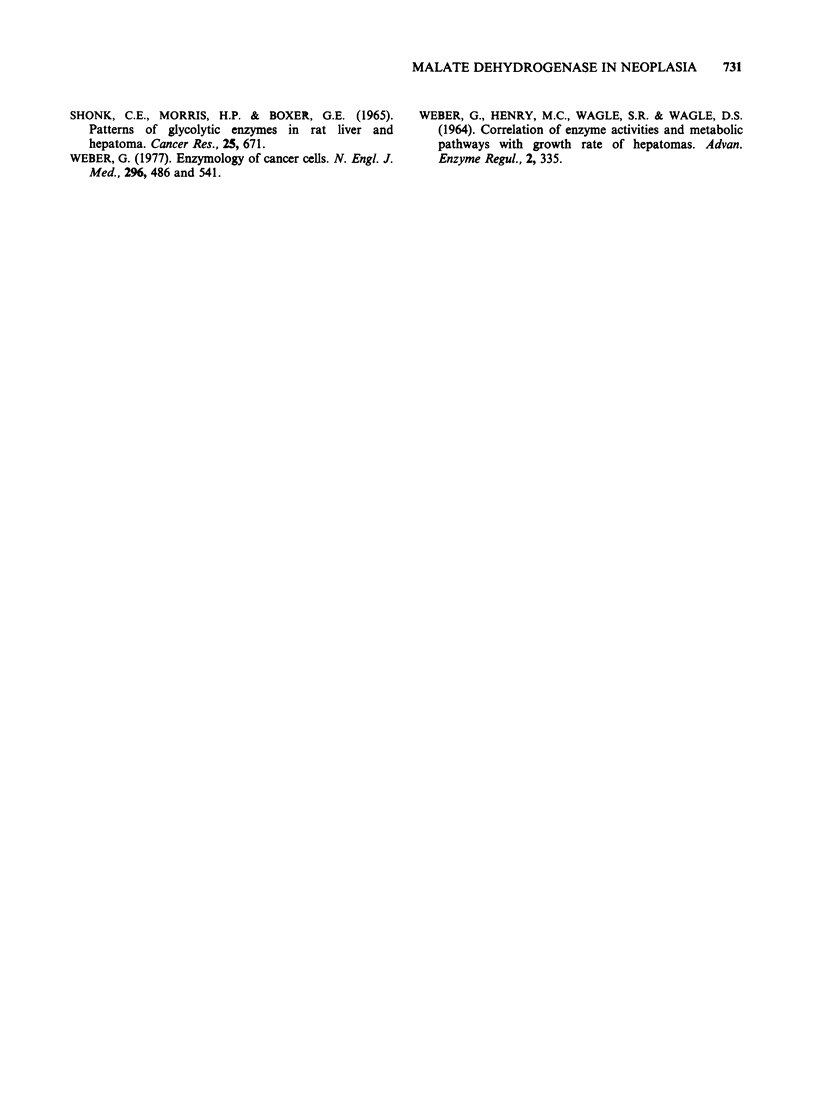# The cytoplasmic malate dehydrogenase in neoplastic tissues; presence of a novel isoenzyme?

**DOI:** 10.1038/bjc.1983.113

**Published:** 1983-05

**Authors:** M. B. Grisham, L. H. Bernstein, J. Everse


*Br. J. Cancer* (1983), **47**, 727–731

### Short communication

### The cytoplasmic malate dehydrogenase in neoplastic tissues; presence of a novel isoenzyme?

M.B. Grisham[1]*, L.H. Bernstein[2]† & J. Everse[1]

[1]*Department of Biochemistry, Texas Tech University Health Sciences Center, Lubbock, TX 79430, and*
[2]*Department of Pathology, University of South Florida, College of Medicine, Tampa, Florida, U.S.A.*

Malate dehydrogenase (MDH, EC 1.1.1.37) catalyzes the reversible reduction of oxaloacetate to malate in the presence of NADH. In eukaryotic cells the enzyme is generally found to be present as two distinct isoenzymes; one form is present in the cellular cytosol and the other is present exclusively in the mitochondria. These 2 isoenzymes form part of a shuttle system (the malate-aspartate shuttle) that functions as the major mechanism for the transportation of reducing equivalents between the cytosol and the mitochondria.

As part of our ongoing studies on the mechansim of action and metabolic function of the malate dehydrogenases (Bernstein *et al.* 1978; Bernstein & Everse, 1978; Bernstein & Grisham 1978), we recently investigated the kinetic properties of the 2 isoenzymes present in rat Novikoff hepatoma tissues. These studies were initiated to evaluate whether or not the enzymes in the malate-asparate shuttle of tumour tissues are structurally and functionally identical to those of normal tissues.

Fresh tumour or liver was homogenized with a glass tissue homogenizer in 0.1 M potassium phosphate buffer, pH 7.5, containing 0.25 M sucrose. The homogenate was centrifuged for 10 min at 10,000 g to remove tissue debris. The supernatant was then centrifuged for 30 min at 20,000 g to obtain a high-speed supernatant that contained the cytoplasmic enzymes. The supernatantant did not contain any isocitrate dehydrogenase activity or transhydrogenase activity and was therefore judged to be free of mitochondrial enzymes. This high-speed supernatant was used without further fractionation for the determination of the cytoplasmic MDH activity. Mitochondria were prepared by resuspending the pellet in 0.1 M phosphate buffer, pH 7.5, containing 0.25 M sucrose and centrifuging the suspension at 600 g. The supernatant was then centrifuged at 20,000 g for 30 min, the precipitate was collected and the washing procedure was repeated once more. Finally, the precipitate was suspended in phosphate buffer and sonicated for 1 min. The resulting solution was used for the assays for the mitochondrial enzyme.

The assays were performed in 0.1 M phosphate buffer, pH 7.0, as described in the legend to Figure 1. Oxaloacetate and NADH solutions were freshly prepared prior to use and kept in ice during the analysis. The homogenates were also kept in ice until the assays were completed. Assays were performed at room temperature with a Beckman Model 24 recording spectrophotometer.

A determination of the $K_M$ values for the mitochondrial and the cytoplasmic enzyme yielded the values listed in Table I. We found that the $K_M$ values of the mitochondrial enzyme from the hepatoma tissue were identical with the values obtained with the enzyme from normal liver mitochondria. The cytoplasmic enzymes also have identical $K_M$ values for the coenzyme; however, the Lineweaver-Burk plots for oxaloacetate were non-identical. Whereas the $K_M$ value for oxaloacetate obtained with the liver enzyme was $\sim 55\,\mu$M, the Lineweaver-Burk plot obtained with the hepatoma enzyme displayed 2 slopes as illustrated in Figure 1. One of the slopes corresponded with a $K_M$ value that is approximately identical to that of the liver enzyme, whereas the other slope yielded a $K_M$ value for oxaloacetate of $\sim 1$ mM.

We interpret these data to indicate that Novikoff hepatoma tissue contains 2 cytoplasmic enzymes that possess MDH activity, one of which closely resembles that present in the rat liver cytoplasm. The other enzyme, having a $K_M$ of $\sim 1$ mM, is not found in normal liver tissue.

The cytoplasmic MDH activity in the hepatoma tissue with the same kinetic properties as the liver enzyme could be due to a contamination of the

Correspondence: J. Everse

*Present Address: Division of Biochemistry, St. Jude Children's Research Hospital, Memphis, TN 38101.

†Present Address: Department of Pathology, United Health Services, Johnson City, NY 13790.

Received 18 November 1982; accepted 9 February 1983

© The Macmillan Press Ltd., 1983

**Figure 1** Lineweaver-Burk plots of the cytoplasmic malate dehydrogenase activity from rat liver (**A**) and rat Novikoff hepatoma tissue (**B**) as a function of oxaloacetate concentration. Assay mixtures contained 0.14 mM NADH, the indicated amount of oxaloacetate and an appropriate amount of the enzyme in 0.2 M phosphate buffer, pH 7.0. Total volume 3 ml. Each point represents the average of 3 determinations if the obtained values were within 5% error. Additional assays were done if a larger deviation was observed.

**Table I** $K_M$ values for the malate dehydrogenase isoenzymes in normal and neoplastic rat tissues

| | *Malate* (*μM*) | *Oxaloacetate* (*μM*) |
|---|---|---|
| Rat Liver: | | |
| mitochondrial MDH | 92 | 40 |
| cytoplasmic MDH | — | 55 |
| Rat Novikoff Hepatoma: | | |
| mitochondrial MDH | 90 | 40 |
| cytoplasmic MDH | — | 55; 1000 |

tumour tissue with normal tissue. To test this possibility, we purified the Novikoff hepatoma cells by growing them in tissue culture using a Delbucco's modified essential medium, and transferring the culture until only neoplastic cells could be detected in the culture. An extract of these cells was subsequently used for the determination of the cytoplasmic MDH activity. The obtained Lineweaver-Burk plot was essentially identical to that shown in Figure 1B. It appears thus that hepatoma cells contain the aberrant cytoplasmic MDH as well as the normal enzyme. The specific activity of the cytoplasmic MDH in rat livers was found to vary from 20–300 μmol NADH oxidized $min^{-1} g^{-1}$ wet tissue. For the hepatoma tumours this value ranged from 250–400 $\mu mol\, min^{-1} g^{-1}$. These values are somewhat higher than those reported by others (Weber *et al.*, 1964; Shonk *et al.*, 1965), which is likely the result of our use of a higher substrate concentration (6.6 mM oxaloacetate). The data indicate, however, that the aberrant enzyme is not present in addition to regular amounts of the normal enzyme, but some of the normal enzyme is replaced by the aberrant enzyme. Using the values for Vmax in Figure 1B, one calculates that the cytoplasmic MDH in the rat hepatoma consists of ~20% of the normal enzyme and 80% of the aberrant enzyme.

To obtain further evidence for the existence of 2 distinct cytoplasmic enzymes, mitochondria-free extracts of a rat Novikoff hepatoma as well as of Novikoff hepatoma cells from tissue cultures were prepared and subjected to electrophoresis on Beckman cellulose acetate membranes at several pH values. A normal rat liver extract was used as a reference. The strips were stained using the tetrazolium staining mixture described by Fine & Costello (1963). Staining was continued for an extended period of time in order to detect any isoenzymes that might be present in small amounts. However, no differences in isoenzyme distribution between the tumour and the normal liver could be detected. We concluded, therefore, that the aberrant MDH is electrophoretically indistinguishable from the normal cytoplasmic MDH.

An extensive search for the presence of the aberrant enzyme in any normal rat tissue yielded negative results. The presence of the unusual enzyme was detected, however, in extracts of foetal rat liver. Our data obtained thus far suggest that the enzyme with the low affinity for oxaloacetate is present during the early foetal stages, but gradually disappears during the gestation period. None of the aberrant enzyme appears to be present in the livers of newborn rats.

We subsequently investigated whether or not the aberrant MDH is present in other rat tumours as well. The same differences in catalytic properties were found when the cytoplasmic MDH activities present in the livers of Sprague-Dawley and Buffalo rats were compared with those of the hepatoma H5123 (Morris minimal deviation) and the hepatoma 19 (ethionine-induced rapidly growing tumour). In these experiments $K_M$ values of ~1 mM were found for oxaloacetate, using the tumour extracts, whereas the $K_M$ values for the liver extracts were 0.05 mM. Similar changes were found in a virus-induced hamster lymphoma.

A cytoplasmic MDH with characteristics similar to those of the aberrant MDH is also present in various human tumours. The various types of tumours thus far investigated that contain the unusual MDH are listed in Table II. The unusual enzyme was absent in extracts of a granuloma and a fibroma. The latter tissues yielded double reciprocal plots with a single slope that closely resembled that obtained with extracts from normal liver tissues. The $K_M$ for oxaloacetate of the cytoplasmic MDH from normal human liver extracts was found to be 40 $\mu$M. Double reciprocal plots of the data obtained from all other tissues listed in Table II yielded 2 slopes as shown in Figure 2B. The $K_M$ value of the aberrant enzyme is again ~1 mM.

The unusual cytoplasmic MDH can also be detected in the serum of animals and patients with neoplastic disease. To demonstrate this, 2 assays were done; one at an oxaloacetate concentration of 0.33 mM and the other at an oxaloacetate concentration of 6.6 mM. Both substrate concentrations are well above the $K_M$ of the normal cytoplasmic MDH and the rate of NADH oxidation should be about independent of the oxaloacetate concentration. Therefore the ratio of the two rates should be close to unity. When such assays were done on the sera of about 20 healthy individuals, values between 0.8 and 1.0 were obtained for the ratios of the two assays. Using sera obtained from patients with neoplastic disease, we obtained ratios between 2.0 and 3.0 (Table III), indicating the presence of an enzyme with a high $K_M$ value for oxaloacetate.

An analysis of serum samples for MDH activity as described could be a useful tool in the early diagnosis of certain malignant growths. In addition, the technique could be used to evaluate the effectiveness of various therapeutic treatments as well as of the surgical removal of a malignancy. Further details concerning these applications will be presented elsewhere.

The question that remains to be answered concerns the nature of the aberrant enzyme. Several possibilities deserve consideration. The first possibility is that the activity represents a hitherto unknown MDH isoenzyme that is only present during embryonic development and in certain neoplasms. The existence of such a novel isoenzyme would explain our present observations. The presence of the isoenzyme can readily be

**Figure 2** Lineweaver-Burk plots of the cytoplasmic malate dehydrogenase activity from human liver (**A**) and human adenocarcinoma tissue (**B**) as a function of oxaloacetate concentration. Assays mixtures were as described in the legend of **Figure 1**.

**Table II** $K_M$ for oxaloacetate in tissue homogenates of human origin

| *Tissue* | $K_M$ *(mM)* |
|---|---|
| Adenocarcinoma (Breast) | 1.0, 1.2 |
| Adenocarcinoma (Colon) | 1.0 |
| Adenocarcinoma (Uterus) | 1.0 |
| Squamous cell carcinoma (Throat) | 0.9 |
| Lymphocytic leukaemia | 1.1 |
| Granuloma | 0.035 |
| Fibroma | 0.048 |
| Liver | 0.04 |

**Table III** $K_M$ for oxaloacetate in sera from cancer patients

| *Tumour* | $K_M$ *(mM)* | *Ratio** |
|---|---|---|
| Lymphocytic leukaemia | 0.9 | 2.0 |
| Adenocarcinoma (Liver) | 1.1 | 2.1 |
| Adenocarcinoma (Colon) | 1.0 | 2.1 |
| Adenocarcinoma (Stomach) | 1.0 | 2.0 |
| Adenocarcinoma (Ovary) | 1.0 | 2.0 |
| Normal | 0.05 | 1.0 |

*Ratio = Reaction velocity at 6.6 mM OAA divided by velocity at 0.33 mM OAA.

demonstrated by its catalytic activity, but is electrophoretically indistinguishable from the native isoenzyme. It is very difficult to see, however, how the presence of a MDH isoenzyme with a very high $K_M$ for oxaloacetate can provide a metabolic advantage to rapidly growing cells (Weber, 1977).

A second possibility is that the activity results from an enzyme that has some non-specific malate dehydrogenase activity. Lactate dehydrogenase would be a likely candidate for this, since it is closely related to MDH in many respects. However, the possibility that lactate dehydrogenase is acting as a malate dehydrogenase isoenzyme can be ruled out on the following observations:

(a) Lactate dehydrogenase activity is electrophoretically distinguishable from MDH activity.
(b) The aberrant activity was undetectable in normal tissues, indicating that the aberrant activity must be present in much higher concentrations in tumour tissues than in normal tissues. We found that the lactate dehydrogenase activity in various tumour tissues tested is not more than 2–5 times higher than that in normal tissues, where it is of course present in appreciable amounts.
(c) The $K_M$ of lactate dehydrogenase for oxaloacetate varies from 1.75 mM at pH 6.0 to 4.9 mM at pH 7.4 (Parker & Holbrook, 1981), which is considerably higher than the value of 1.0 mM at pH 7.0 that we found for the aberrant enzyme.

A third possibility is that the oxaloacetate is partially converted to pyruvate by a decarboxylase present in the tumour tissues. The pyruvate thus formed would readily be reduced by the lactate dehydrogenase present in the extracts and thus contribute to the observed decreases in NADH in our assays. The fact that the $K_M$ values that we obtained for the MDH from normal tissues (and for tumour tissues at low oxaloacetate concentrations) are similar to the values obtained by others (Englard, 1969; Kitto & Kaplan, 1966) with highly purified MDH indicates that significant decarboxylation of the oxaloacetate is not a problem at the low oxaloacetate concentrations. In fact, if the aberrant activity resulted from the decarboxylation of oxaloacetate to pyruvate, we would in effect have measured the $K_M$ of the decarboxylase for oxaloacetate as being 1.0 mM, since the $K_M$ of lactate dehydrogenase for pyruvate is very low (Everse & Kaplan, 1973). The fact that the oxaloacetate concentration in tumour cells as well as normal cells is far below 1 mM again raises a question as to the possible metabolic advantage that such a decarboxylase would provide for the tumour cell.

It is clear that the question addressed by this article must remain unanswered until the enzyme that promotes the aberrant activity has been purified and fully characterized. The finding of an isoenzyme with an increased $K_M$ in tumour tissues would not be unique, however. Weber *et al.* (1964) showed that the phosphoglucomutase in rapidly growing hepatomas has a $K_M$ for glucose-1-P that is 3–6 times higher than the $K_M$ value found for the normal (liver) enzyme. It is thus possible for certain tumour cells to synthesize enzymes different from those produced by normal cells, thus reflecting changes in genetic expression. More detailed information concerning such changes in enzyme characteristics could be useful both for diagnostic and for chemotherapeutic purposes.

This work was sponsored by the Milheim Foundation for Cancer Research during the early experimental stages, by the American Heart Association, Florida Chapter and by a grant from the Institute of Biomedical Research, TTUHSC. Many helpful suggestions were provided by Dr. H. Sidransky, Department of Pathology, George Washington Unviersity School of Medicine.

### References

BERNSTEIN, L.H. & EVERSE, J. (1978). Studies on the mechanism of the malate dehydrogenase reaction. *J. Biol. Chem.*, **253**, 8702.

BERNSTEIN, L.H. & GRISHAM, M.B. (1978). Kinetic determination of malate dehydrogenase isoenzymes. *J. Mol. Cell. Cardiol.*, **10**, 931.

BERNSTEIN, L.H., GRISHAM, M.B., COLE, K.D. & EVERSE, J. (1978). Substrate inhibition of the mitochondrial and cytoplasmic malate dehydrogenases. *J. Biol. Chem.*, **253**, 8697.

ENGLARD, S. (1969). Extramitochondrial L-malate dehydrogenase of beef heart. *Methods Enzymol.*, **13**, 123.

EVERSE, J. & KAPLAN, N.O. (1973). Lactate dehydrogenases: Structure and function. *Adv. Enzymol.*, **37**, 61.

FINE, I.H. & COSTELLO, L.A. (1963). The use of starch gel electrophoresis in dehydrogenase studies. *Methods Enzymol.*, **6**, 958.

KITTO, G.B. & KAPLAN, N.O. (1966). Purification and properties of chicken heart mitochondrial and supernatant malic dehydrogenase. *Biochemistry*, **5**, 3966.

PARKER, D.M. & HOLBROOK, J.J. (1981). The oxaloacetate reductase activity of vertebrate lactate dehydrogenase. *Eur. J. Biochem.*, **13**, 1101.

SHONK, C.E., MORRIS, H.P. & BOXER, G.E. (1965). Patterns of glycolytic enzymes in rat liver and hepatoma. *Cancer Res.*, **25**, 671.

WEBER, G. (1977). Enzymology of cancer cells. *N. Engl. J. Med.*, **296**, 486 and 541.

WEBER, G., HENRY, M.C., WAGLE, S.R. & WAGLE, D.S. (1964). Correlation of enzyme activities and metabolic pathways with growth rate of hepatomas. *Advan. Enzyme Regul.*, **2**, 335.